# Automated Modular Magnetic Resonance Imaging Clinical Decision Support System (MIROR): An Application in Pediatric Cancer Diagnosis

**DOI:** 10.2196/medinform.9171

**Published:** 2018-05-02

**Authors:** Niloufar Zarinabad, Emma M Meeus, Karen Manias, Katharine Foster, Andrew Peet

**Affiliations:** ^1^ Institute of Cancer and Genomic Sciences University of Birmingham Birmingham United Kingdom; ^2^ Birmingham Children Hospital NHS Trust Birmingham United Kingdom; ^3^ Physical Sciences of Imaging in Biomedical Sciences Doctoral Training Centre University of Birmingham Birmingham United Kingdom

**Keywords:** clinical decision support, real-time systems, magnetic resonance imaging

## Abstract

**Background:**

Advances in magnetic resonance imaging and the introduction of clinical decision support systems has underlined the need for an analysis tool to extract and analyze relevant information from magnetic resonance imaging data to aid decision making, prevent errors, and enhance health care.

**Objective:**

The aim of this study was to design and develop a modular medical image region of interest analysis tool and repository (MIROR) for automatic processing, classification, evaluation, and representation of advanced magnetic resonance imaging data.

**Methods:**

The clinical decision support system was developed and evaluated for diffusion-weighted imaging of body tumors in children (cohort of 48 children, with 37 malignant and 11 benign tumors). Mevislab software and Python have been used for the development of MIROR. Regions of interests were drawn around benign and malignant body tumors on different diffusion parametric maps, and extracted information was used to discriminate the malignant tumors from benign tumors.

**Results:**

Using MIROR, the various histogram parameters derived for each tumor case when compared with the information in the repository provided additional information for tumor characterization and facilitated the discrimination between benign and malignant tumors. Clinical decision support system cross-validation showed high sensitivity and specificity in discriminating between these tumor groups using histogram parameters.

**Conclusions:**

MIROR, as a diagnostic tool and repository, allowed the interpretation and analysis of magnetic resonance imaging images to be more accessible and comprehensive for clinicians. It aims to increase clinicians’ skillset by introducing newer techniques and up-to-date findings to their repertoire and make information from previous cases available to aid decision making. The modular-based format of the tool allows integration of analyses that are not readily available clinically and streamlines the future developments.

## Introduction

Magnetic resonance imaging (MRI) is a fast-growing clinical imaging modality and has become the modality of choice for the evaluation of disease and treatment management across multiple therapeutic areas. It has increasingly been used in oncology, central nervous system diseases, musculoskeletal disorders, and cardiovascular disease due to its superior soft-tissue imaging capabilities, lack of ionizing radiation, and noninvasive nature [1-4].

MRI technology constantly advances with new magnetic resonance applications being pioneered, investigated, mainstreamed, and added to clinical applications and capabilities. Nevertheless, clinical interpretation remains largely by qualitative expert review. In addition, new advanced and computationally intensive medical quantitative image analysis techniques are constantly being developed and validated. These techniques have allowed the discovery of specific biomarkers of both disease and treatment response and have exposed clinicians to new information in a computable format [5-8]. However, the growing and versatile amount of magnetic resonance–derived information can form an insurmountable obstacle to the individual clinician; in particular, the use of quantitative MRI biomarkers requires further improvement in accessibility and presentation to aid decision making [9].

In the past decade, clinical decision support (CDS) systems have increasingly gained attention, and the routine uptake of these intelligent systems is becoming more common [10-17]. Introduction of CDSs has provided clinicians and health care investigators with a platform for extraction of relevant information to aid decision making, prevent errors, and enhance health care. CDSs include a range of options from computerized alerts, reminders, and clinical guidelines to diagnostic support and clinical workflow through computer-assisted diagnosis tools (CAD) [18-26]. There are several clinically implemented or research-based CADs available for medical image analysis [23,26-29]. However, majority of them lack at least one of the following: (1) a user-friendly graphical interface to be used by clinicians in their clinical routine; (2) system performance is often not compared with radiologist diagnosis in the absence of the tool or when the tool is utilized; (3) are not MRI based; (4) are designed for one particular disease; and (5) are just a single postprocessing tool or analysis algorithm, which also provides a likelihood for a disease and does not offer decision support for the clinicians (ie, in form of only providing additional structured information for comparison with available other relevant diagnosis). These types of solutions have shown to suffer from high false positives [9].

Availability of a user-friendly and flexible MRI CAD that encompasses a variety of medical image analysis techniques and postprocessing methods and can act as a CDS could facilitate the uptake of new advanced magnetic resonance techniques in the real-time clinical setting; it could also allow health care investigators to interrogate their data in a scientifically informative and convenient manner to determine a robust and efficient diagnosis. The aim of this study was to design, develop, and evaluate a medical image region of interest analysis tool and repository (MIROR) platform for conventional magnetic resonance data aimed at improving clinical performance through the provision of real-time diagnostic support for clinicians.

To the best of our knowledge, there is only the International Network for Pattern Recognition of Tumours Using Magnetic Resonance Decision Support System validated and available for the analysis of magnetic resonance spectroscopy (MRS) data [30]. However, this CDS is developed for diagnosing and grading adult brain tumors and is based on MRS only. There is no CDS for both MRI and MRS analysis with a robust user interface for clinical routine use that is capable of creating and updating a validated repository for different diagnostic problems.

## Methods

### Clinical Decision Support System Design

Features available in the presented version of the MIROR are (1) a clinician-friendly graphical user interface; (2) measurement of morphologic properties such as size, shape, volume, length dimensions, and center-of-mass location of the region of interest (ROI); (3) an integrated magnetic resonance diffusion-weighted imaging (DWI) analysis application based on intravoxel incoherent motion (IVIM) model; (4) statistical data analysis of the ROI overlaid on standard MRI images (such as T1-weighted and T2-weighted scans or the advanced quantitative maps) to provide decision support in forms of comparison with other differential diagnosis and several different image volumes to aid diagnosis and determination of prognosis; and (5) a self-archiving repository of the extracted data and features. Availability of the latter 2 options in combination with the first 3 will move the designed tool from a CAD toward becoming a CDS for MRI data. MIROR also allows investigators to further grow, advance, and combine different analysis techniques and types of imaging sequences to extend the tool to a more sophisticated decision support, dependent on their individual center’s needs to better inform diagnosis. MIROR’s self-archiving, evolving repository is the core of its decision support. This unique feature of the MIROR distinguishes it from pervious CADs and CDSs. First, the repository’s continuous development allows for improvement in the predication accuracy for the available biomarkers and disease in the database; second, it permits provision of a decision support system compatible to additional disease types by means of importing and appending the repository.

**Figure 1:**
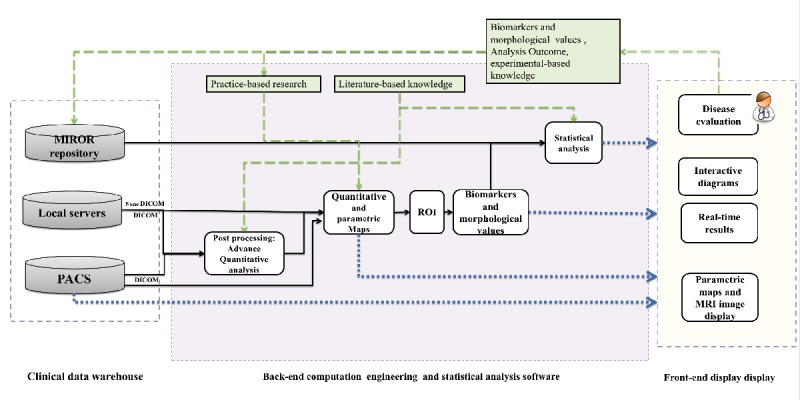
Flow diagram showing hierarchical structure of the medical image region of interest analysis tool and repository (MIROR) infrastructure. Dashed blue lines indicate direct connection of the module output to the front-end display, solid lines are the connections between internal clinical decision support (CDS) modules, and green dashed lines represent the feedback system to the repository. PACS: picture archiving and communication system; ROI: region of interest; MRI: magnetic resonance imaging. MRI: magnetic resonance imaging; DICOM: digital imaging and communications in medicine.

We used a modular and open architecture design [23] in the design and implementation of MIROR to be able to adapt to the constant increase and development in the MRI sequences; it will also make room for consequent advances in the related analysis applications and allow future development of additional new workflows. Additionally, we used Mevislab software (v. 2.7.1, MeVis AG- Fraunhofer-MEVIS) [31,32], a research-based rapid prototyping platform for medical image processing, for development of the MIROR to achieve the latter. Post processing, quantitative and statistical analysis functionalities embedded in MIROR were either developed using Python (v. 2.7, embedded within Mevislab) or were imported from the Mevislab library. The MIROR repository was developed using Python. Each individual independent module of MIROR was developed, evaluated, and tested by different groups within the team in their own life cycle and schedules before addition to the final product. A hierarchical structure of MIROR infrastructure is represented in Figure 1. MIROR self-archiving, evolving repository is the core of its decision support. This feature of the MIROR distinguishes it from pervious computer-assisted diagnosis tools (CADs) and CDSs.

**Figure 2:**
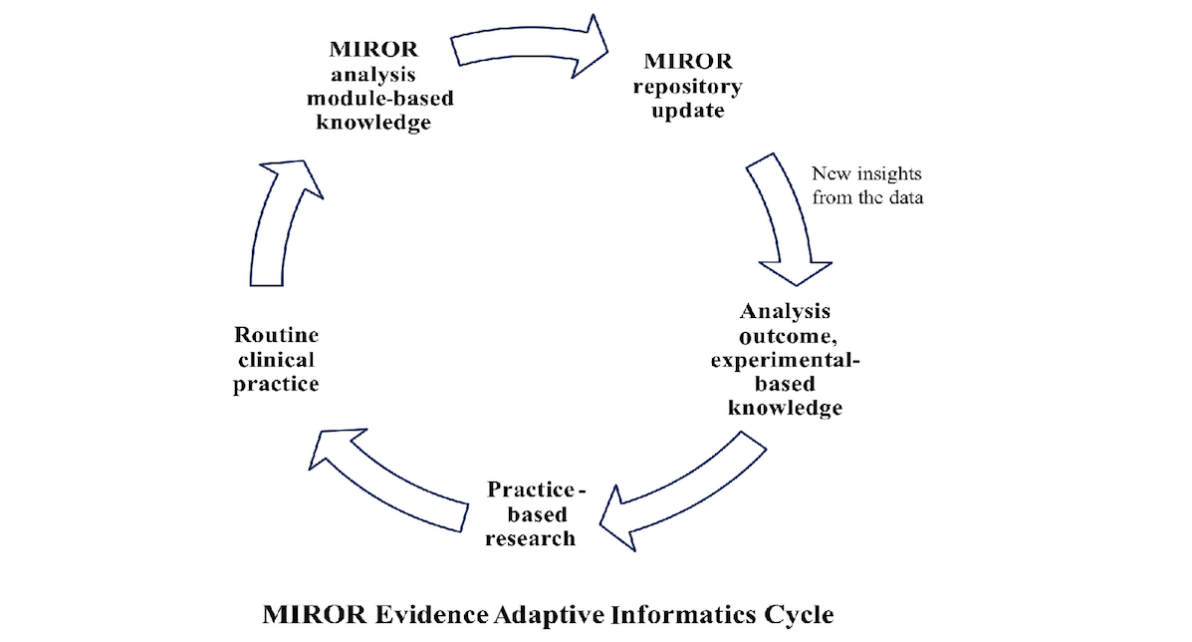
Medical Image Region of interest analysis tool and Repository (MIROR) evidence-adaptive protocol. An evidence-adaptive approach was utilized in the design of the MIROR by utilizing its knowledge base to derive from and reflect on the most up-to-date evidence from the research literature and practice-based sources.

Based on the recommendations of the American medical informatics association [33], an evidence-adaptive approach was employed in the design of the MIROR by utilizing its knowledge base to derive from—and reflect on—the most up-to-date evidence from the research literature and practice-based sources [34]. The statistical and quantitative analysis module embedded in MIROR are developed based on the literature and local practice-based research and will continue to update in future releases. MIROR is an evolving database of available diagnosis data gathered from routine clinical practice. Outcomes of this repository data analysis can inform future clinical investigations, reflect on the clinical practice, and consequently impact on the MIROR statistical and quantitative analysis module. Conversely, practice-based experience can inform the choice of MRI sequence and parametric maps to be used for analysis and clinical evaluation (Figure 2).

MIROR can import all file formats supported by the National Library of Medicine Insight Segmentation and Registration Toolkit, such as digital imaging and communications in medicine (DICOM) files, Neuroimaging Informatics Technology Initiative files, JPEG as well as text files, and comma spreadsheets. Built-in Mevislab modules and Python were used to create this functionality. Using the module raw images, postprocessed quantitative maps and data files can be imported from the clinical data warehouses, such as hospital picture archiving and communication system (PACS), the local servers, or MIROR for future analysis and visualization (Figure 1).

Comparing the MIROR architecture with previously developed CDSs with a clinical data base and domain expert knowledge base, MIROR does not connect to hospital electronic health record (EHR) system or any Internet-based database or medical knowledge representations or guidelines [10,35,36]. Having said so, one should note that imaging data can be imported to MIROR through connection to hospital PACS and therefore can be considered as a semi-integrated CDSs [37]. Moreover, currently available active and robust CDSs benefit from EHR data with very large and historical dataset that changes continuously and contains hidden knowledge. MIROR was designed based on a similar architecture applied to a repository containing a constantly updated independent database. The updating of the database allows the advanced MRI biomarkers to be revised whenever new data are available.

One of the main strengths of MIROR is its ability to allow for integration of new advanced and computationally intensive quantitative analyses that are not readily available to be used in routine clinical practice under the Advance Quantitative analysis module (Figure 1). In this study, the analysis of multi b-value (b=diffusion weightings) magnetic resonance DWI is embedded in MIROR. The analysis was developed using the well-established and not clinically available intravoxel incoherent motion (IVIM) model, which has been shown to have clinical value in many different tumor types [38-40] as well as in other pathologies [41,42]. Although IVIM provides a similar measure to clinically available apparent diffusion coefficient (ADC), derivation of the additional parameters allows the separation of the perfusion contribution from the true diffusion, resulting in a greater insight to the underlying tissue microenvironment [43-45].

This analysis allows the computation of tissue diffusion coefficient (IVIM-*D*), pseudo-diffusion coefficient (IVIM-D*), and perfusion fraction (IVIM-*f*) [46]. However, as a relatively new analysis method, it is currently not available as part of the scanner software packages. The MIROR IVIM module, which is developed using python programming, loads the raw DWI DICOM file and based on it determines whether the IVIM analysis is feasible, based on the number of the b values. The output from the module is the IVIM parameter maps, which can then be used for further ROI-based statistical analysis.

The MIROR-embedded statistical analysis module provides specialists with instruments for (1) analyzing and interpreting individual patient MRI data and (2) comparing it with results of previous cases directly from the MIROR-evolving repository via powerful statistical techniques to future inform the investigation (Figure 1). MIROR provides statistical analysis of the advanced parametric maps or the standard MRI sequence image’s ROI (ie, diffusion maps produced in MIROR, other imported advance quantitative maps, or T1-weighted and T2-weighted scans). It measures the volume of the defined ROI, creates a histogram for it, performs statistical analysis on the ROI values, and extracts and stores histogram parameters such as entropy, median, mean, different quantiles, skewness, and kurtosis in its repository. Unlike previously reported CAD and CDS tools, using MIROR the medical expert has multiple options, including (1) selection of the population of interest from the repository to work only with data from a specific condition, (2) choosing the MRI biomarker/variable (eg, diffusion, perfusion), and (3) the statistical variable of interest from a complete set of basic and advanced features that cover both clinical and research needs (basic statistics mean, median, variance, standard deviation, quantile, histogram analysis, etc).

Note that MIROR is a nonregion-specific MRI CDS, and its novel quantitative image analysis and statistical analysis modules are designed to analyze any region of the body and aid in resolving different demanding diagnostic problems.

The frontend of the MIROR is a clinician’s user-friendly graphical interface that displays MRI images as well as quantified parametric maps and allows clinicians to define their ROI. It also provides real-time morphological and statistical results for compassion with the repository data so as to aid clinical evaluation of the disease.

### Medical Image Region of Interest Analysis Tool and Repository Application to Pediatric Tumor Evaluation

MIROR is currently being developed, evaluated, and used at Birmingham Children’s Hospital to determine its role in facilitating noninvasive diagnosis in children presenting with solid body tumors in clinical practice.

Solid masses in children represent a diagnostic dilemma, as neoplastic and non-neoplastic lesions can appear similar on conventional imaging. Although in some cases the clinical history and physical examination findings indicate a likely diagnosis, the majority of cases require further evaluation with MRI to assess the extent of the lesion and make a specific diagnosis. It is often difficult to determine whether a lesion is benign or malignant or identify specific tumor type based on conventional MRI alone.

**Table 1:** Body tumor patient cohort demographics.

Tumor	Median age (range)	Gender	Diagnosis	Patients, n
Benign	3.63 (0.03-14.22)	Female=6; Male=7	Liver hemangioma	1
			Ganglioneuroma	4
			Hematocolpos	1
			Lipoma	1
			Infantile myofibromatosis	1
			Mesoblastic nephroma	2
			Hematocolpos	1
			Vascular malformation	1
			Ovarian immature teratoma	1
Malignant	3.94 (0.03-11.82)	Female=16; Male=21	Clear cell sarcoma of kidney	1
			Ewing's sarcoma	1
			Germ cell tumor	1
			Hepatoblastoma	4
			Neuroblastoma	11
			Osteosarcoma	1
			Rhabdoid tumor	2
			Rhabdomyosarcoma	3
			Wilms tumor	13

This study evaluated the impact of information provided by MIROR in aiding clinicians to distinguish between benign and malignant solid body pediatric tumor types using DWI. Accuracy testing involved examination of MIROR for a cohort of real patient cases with recent visits to Birmingham Children’s Hospital and comparison of the MIROR outcome with the radiologist’s initial opinion and final diagnosis derived based on the opinion of the clinical multidisciplinary team of experts together with pathology.

### Magnetic Resonance Imaging Data

The body tumor patient cohort studied consisted of children (aged 0-16 years) with solid tumors, undergoing diagnostic MRI with multi b-value DWI at Birmingham Children’s Hospital from 2012 to September 2016. A total of 48 children were enrolled, of whom 37 had malignant tumors and the rest had benign tumors. Details of the malignant and benign body tumors along with patients’ demographics are presented in Table 1.

We performed the MRI on a Siemens Avanto 1.5 T MRI scanner (Siemens Healthcare, Erlangen, Germany). The diffusion-weighted MRI protocol used an echo-planar imaging sequence in an axial acquisition plane with a field-of-view (FOV) 221 to 350 × 172 to 317 mm^2^, matrix size 122 to 192 × 128 to 192, slice thickness of 5 mm, and in-plane resolution of 1.56 × 1.56 mm^2^. For all subjects, 6 b-values: 0, 50, 100, 150, 600, and 1000 s/mm^2^ were acquired in 3 orthogonal directions with TR/TE=3200 to 9900/92 ms and number of averages=3. The signal to noise of the MRI dataset was approximately 30 (SD 10) for b1000 and 60 (SD 10) for b0 images.

Depending on their ability to cooperate, children were awake, sedated, or under general anesthesia. MRI acquired with different diffusion weightings (b-values) was used to compute ADC (computed from b_0_ and b_1000_), IVIM-*D*, IVIM-D*, and IVIM-*f* maps. These values are a quantitative measure of diffusion related to tissue cellularity [45,47] and can be useful for tumor characterization. Clinicians currently only use ADC maps in a qualitative manner to help tumor characterization, commenting on restriction of diffusion as a possible marker of malignancy. Advanced quantitative diffusion parameters (ie, IVIM-*D*, IVIM-D*, and IVIM-*f*) and means for direct and real-time statistical analysis of these variables are unavailable clinically, despite the growing body of evidence for their potential value in noninvasive diagnosis.

### Discrimination Between Benign and Malignant Tumor Types

To discriminate between malignant and benign tumors, the authors made use of a leave-one-out cross-validation method combined with a {displaystyle C_{30}^{100}approx 3times 10^{25}.} threshold-based classification approach to determine the potential of individual parameters determined by MIROR. To achieve this, one case of the cohort was assigned in turn as the validation case, with the remaining ones used for training to inform the outcome. The selected validation case was then iteratively changed until all cases had been evaluated exactly once. On the basis of the threshold approach, if the value of the case under study lay within 1 standard deviation of the mean of a statistical parameter of the training tumor group (ie, benign or malignant), it was assigned to that particular group. However, if the value of the parameter under study fell within both tumor group regions, we made use of second-adjusted classification based on distance from the mean of the statistical parameter of the groups in the training set. To further evaluate the significance of the statistical parameters and information provided by MIROR, k-nearest neighbor (KNN) and support vector machine (SVM) pattern recognition techniques, followed by leave-one-out cross-validation, were utilized to test the accuracy of derived data in distinguishing between tumor types, using all parameters as classification features. KNN was chosen for its simplicity and performance on basic recognition problems; it has been a ubiquitous classification method with good scalability. SVM outperforms conventional pattern recognition methods, especially when the number of training data is small and number of input variables is large [48].

To account for the data skewness and imbalanced distribution of the 2 groups, synthetic minority oversampling technique has been used to allow for building a larger decision region that contains nearby instances of the minority class [49] when KNN and SVM are used.

Feature selection was performed before classification by means of calculating the significance level of the histogram derived parameters between the tumor groups. Then, benign and malignant tumors’ histogram permanents (ie, Median; 2nd, 5th, 10th, 15th, 25th, 75th, 85th, 90th, and 98th centile values; kurtosis; skewness; and entropy) were compared using the nonparametric Mann-Whitney U test. The authors used the Bonferroni correction method. Parameters showing a significant difference between the 2 groups were used for classification. All statistical analyses were performed using SPSS Statistics (v. 23, Chicago, Illinois) software.

All patients were consented for research to the UK Children’s Cancer and Leukemia Group, Functional Imaging Group database, a UK National Health Service Research Ethics committee-approved study (Reference number 04/MRE04/41, Health Research Authority East Midlands—Derby, UK, Ethical Review Board Chair, Dr Peter Korczak). Informed participation and publication consent was given by parents/guardians.

MIROR is aimed at improving clinical practice through the provision of real-time diagnostic support. To ensure achievement of the latter, each individual application and module has been tested in such an environment by allowing clinicians to interrogate it about the most important clinical questions and provide feedback. We used an iterative process of design, testing, and revision of the MIROR by a diverse team, including medical informatics experts, clinical content experts, and end users to ensure reliable translation of the tool to clinical practice. Experts in MRI and medical imaging, including PhD researchers specializing in MRI and data processing, a physician, and a senior consultant radiologist pilot tested MIROR iteratively during the development and refinement of the tool.

## Results

**Figure 3:**
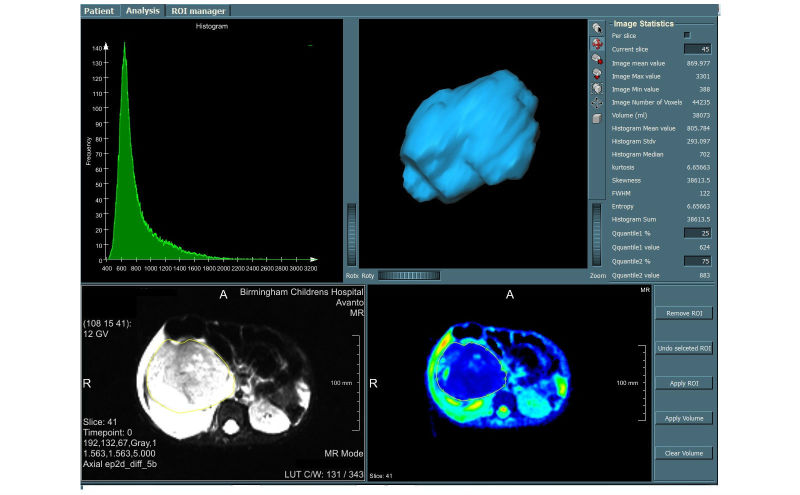
Medical image region of interest analysis tool and repository (MIROR) user-interface patient view. This figure represents data for a malignant tumor case. Here, the region of interest (ROI) is drawn on a high-resolution image and overlaid on the corresponding parametric apparent diffusion coefficient (ADC) map. Measurement of morphologic properties of the ROI, zooming, scaling, rotating of the estimated object surface, and histogram analysis of the overlaid ROI on voxel-by-voxel parametric maps, is supported to enhance quality assessment.

**Figure 4:**
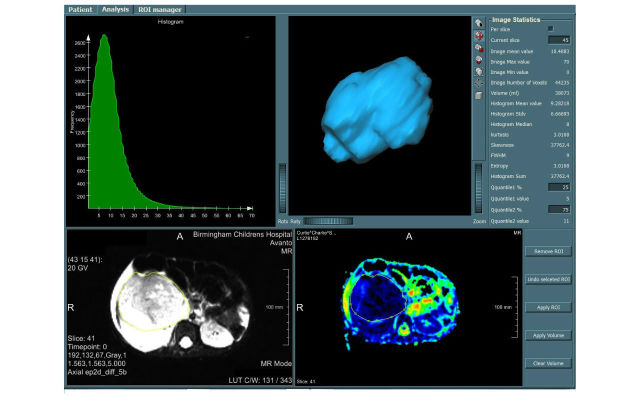
Medical image region of interest analysis tool and repository (MIROR) user-interface intravoxel incoherent motion (IVIM) maps tabs. This figure represents data for a malignant tumor case. Here, the region of interest (ROI) is drawn on a high-resolution image and overlaid on the corresponding parametric map IVIM-D and IVIM-f. Measurement of morphologic properties of the ROI, zooming, scaling, rotating of the estimated object surface and histogram analysis of the overlaid ROI on voxel-by-voxel parametric maps, is supported to enhance quality assessment.

MRI datasets and ADC produced by the scanner were imported to MIROR from a local PACS. An ROI was drawn around the entire solid tumor for each case on a high-resolution image by one clinician, which was then checked by another (KM and KF) before being transferred to a matched parametric map (eg, ADC and IVIM maps). The entire tumor volume, including cystic and necrotic areas, was included in the ROI to determine representative data for heterogeneous tumors [50]. A histogram of the drawn ROI was constructed; the mean, median, 2nd-98th percentile values, skewness, kurtosis, and entropy of the histograms were calculated for all tumors, recorded, and stored on a database. Figure 3 demonstrates a screenshot of MIROR for all of the described stages. Advance IVIM parametric maps D and f are also shown in Figure 4.

**Figure 5:**
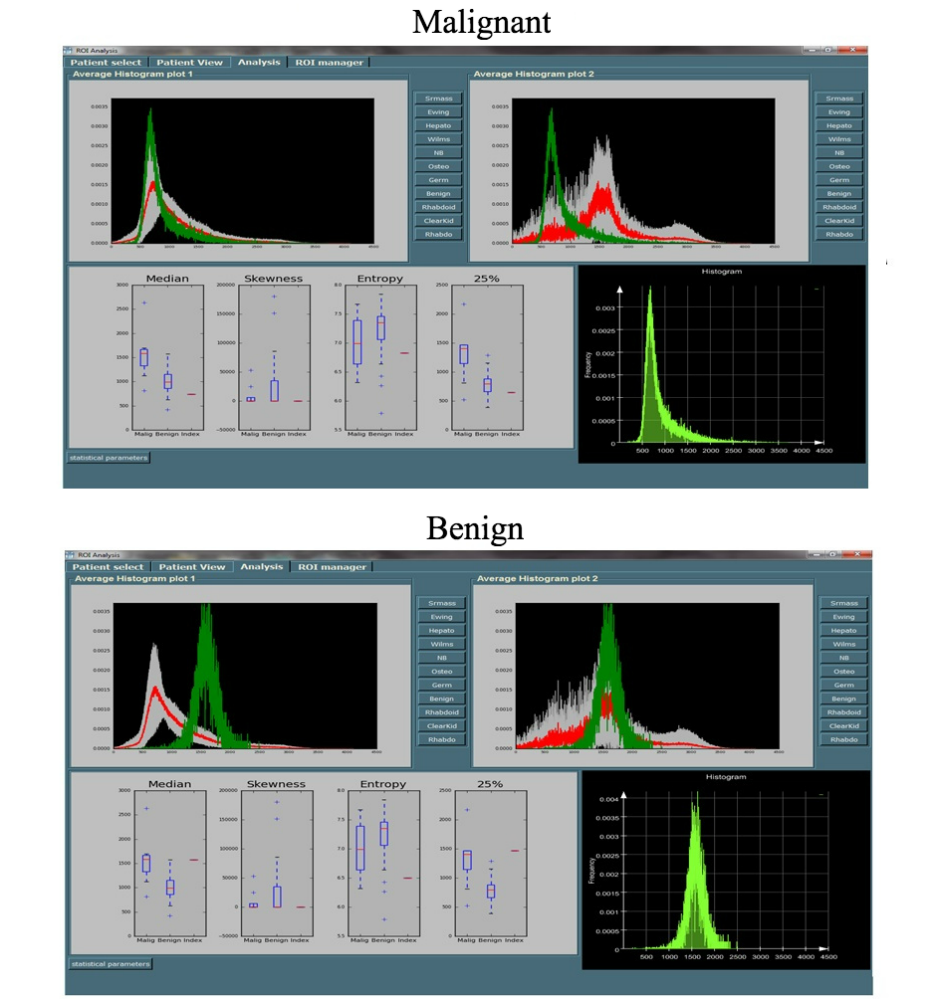
Medical image region of interest analysis tool and repository (MIROR) user-interface analysis tab. This figure represents MIROR use as a decision support system for benign and malignant cases. Here, the green histogram line represents the index case under examination; the red line and gray area represent the mean and standard deviation of the selected tumor group for comparison, respectively. The box plot compares median, skewness, entropy, and 25th percentile values of the index case with the tumor types in the database.

Figure 5 represents an example of MIROR’s decision support module for evaluating benign and malignant cases.

To establish differences between the malignant and benign lesions histogram parameters, the authors compared the histogram-derived parameters in the repository. Analysis showed apparent differences between malignant and benign tumors, with lower ADC values and higher skewness and kurtosis in malignant lesions. There was no significant difference between 85th (*P*=.12), 90th (*P*=.22), 95th (*P*=.82), or 98th centile (*P*=.41) ADC values between benign and malignant tumors (Table 2). Malignant tumors demonstrated statistically significantly lower mean (*P*=.03), median (*P*=.005), 2nd (*P*=.04), 5th (*P*=.02), 10th (*P*=.01), 15th (*P*=.005), 25th (*P*=.004), and 75th centile (*P*=.03) ADC values, higher kurtosis (*P<*.01), more positively skewed histograms (*P*<.001), and higher entropy (*P*=.03). These results are in agreement with similar studies published for adults [51-53].

The feasibility of MIROR to provide distinctive surplus information, which would further aid diagnosis, was evaluated using histogram-derived parameters with the statistically significant differences between the 2 groups. The accuracy of individual statistical parameters to discriminate between specific benign or malignant tumors is presented in Table 3. Due to the high correlation between the ADC centiles, only 15% and 75% centiles were used for classification.

**Figure 6:**
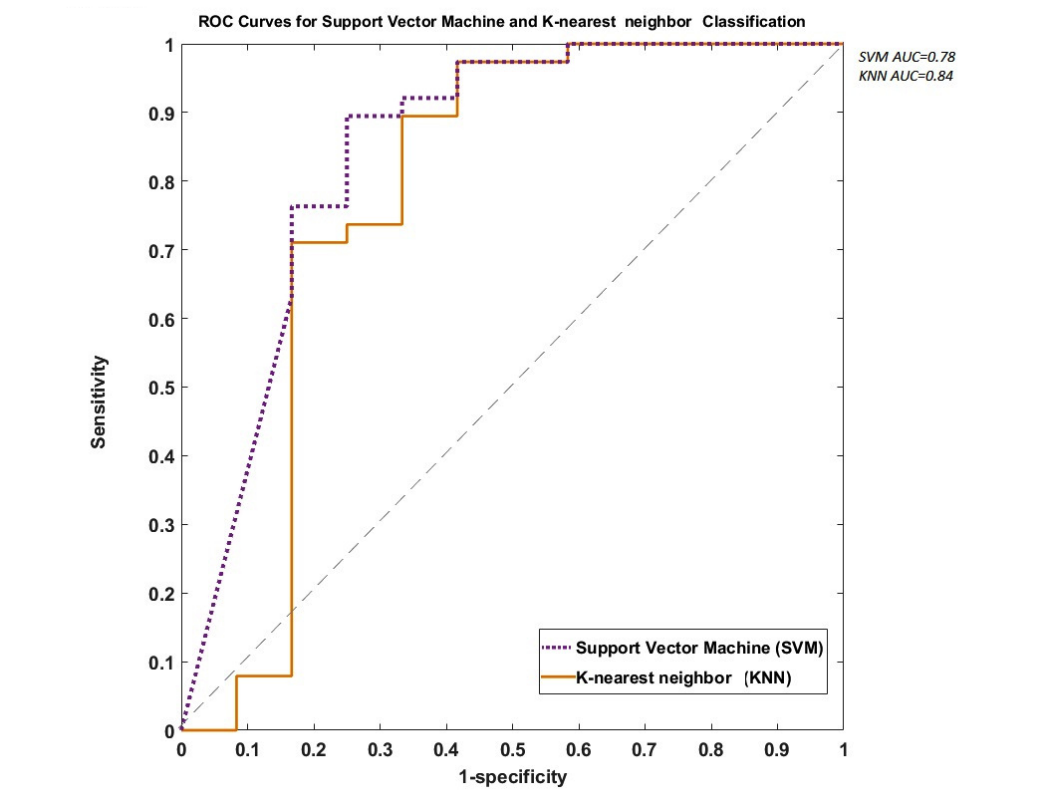
Receiver operating characteristic (ROC) curves of support vector machine and k-nearest neighbor (KNN) in discriminating benign from malignant tumors using medical image region of interest analysis tool and repository (MIROR)-derived parameters. Area under the curve (AUC) was 0.78 for support vector machine (SVM) and 0.84 for KNN.

In the studied cohort, skewness, kurtosis, entropy, different percentiles, mean, and median provided important distinctive additional information, which was not available by using qualitative approaches only. Using kurtosis, entropy and 15th percentile for threshold-based classification, 100% of malignant cases were correctly assigned. Mean, median, and skewness had an accuracy of 0.97 in classifying malignant cases. In the benign category, kurtosis, entropy, and 75th percentile achieved full accuracy. Mean and median had an accuracy of 0.91 in classifying benign tumors. Overall, kurtosis and entropy had the highest sensitivity and specificity (sensitivity=1, specificity=1) in discriminating between benign and malignant tumors. Note that the threshold classification was performed based on a single feature input and a 2-step classification process, with a less strict rule in second layer to reclassify the cases in the ambiguous group. Using all of the above extracted features, more advanced pattern recognition techniques, and 10-fold cross-validation, an accuracy of 0.89 (sensitivity=0.97, specificity=0.5, area under the curve [AUC]=0.78) and 0.93 (sensitivity=0.97, specificity=0.58, AUC=0.84) was obtained by SVM and KNN, respectively. Figure 6 shows a comparison of the classifiers using 10-fold cross-validation receiver operating characteristic analysis.

**Table 2:** Comparison of apparent diffusion coefficient (ADC) histogram parameters between malignant and benign pediatric tumors using the Mann-Whitney *U* test.

Parameters	*P* value
Mean	.03^a^
Median	.005^a^
Kurtosis	<.01^a^
Skewness	<.01^a^
Entropy	.03^a^
2% percentile	.04^a^
5% percentile	.02^a^
10% percentile	.01^a^
15% percentile	.005^a^
25% percentile	.004^a^
75% percentile	.03^a^
85% percentile	.12
90% percentile	.22
95% percentile	.82
98% percentile	.41

^a^Statistical significance *P*<.05.

**Table 3:** Accuracy of individual statistical parameters along with sensitivity and specificity of the analysis obtained by medical image region of interest analysis tool and repository (MIROR) to discriminate between benign or malignant tumors using 2-step threshold classifications.

Marker	Mean	Median	Kurtosis	Skewness	Entropy	15% percentile	75% percentile
Malignant	1098 (SD 295)	996 (SD 262)	2.1 (SD 0.09)	0.02 (SD 0.004)	7.1 (SD 0.42)	710 (SD 201)	1319 (SD 329)
Benign	1443 (SD 462)	1442 (SD 511)	2.031 (SD 0.11)	0.0007 (SD 0.01 )	6.85 (SD 0.4)	1072 (SD 406)	1683 ( SD 538)
Sensitivity	0.94	0.97	1	0.97	1	1	0.97
Specificity	0.91	0.91	1	0.58	1	0.83	1
Accuracy	0.932	0.9455	1	0.6955	1	0.9165	0.987

**Figure 7:**
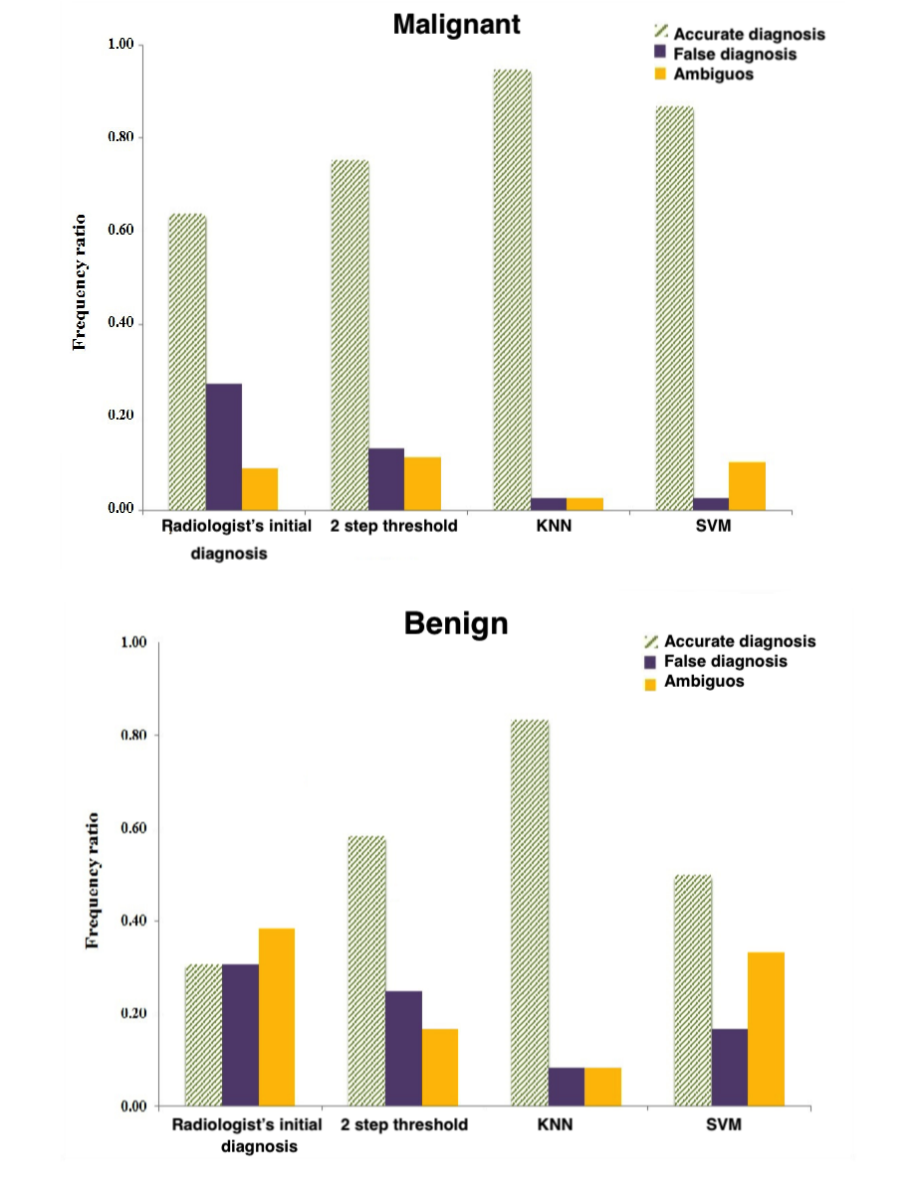
Radiologist’s initial diagnosis compared with final diagnosis after histopathology for different tumor types, along with the comparison between Medical Image Region of interest analysis tool and Repository (MIROR) performance evaluated by support vector machine (SVM), k-nearest neighbor (KNN), and 2-step threshold classification methods.

To further evaluate MIROR in terms of its added clinical value, radiologist initial diagnosis from the first MRI scans was compared with the final diagnosis obtained from histopathology, and the outcome of the 3 statistical analyses based on MIROR provided information for classifying the tumor types for this cohort of patients (Figure 7). For the 2-step thresholding classification, outcome of the first classification layer is presented to include the ambiguous group. The ambiguous groups for KNN and SVM were identified by thresholding their predication probabilities at above 0.8 and above 0.5 for the accurate and ambiguous assignment of cases, respectively.

A higher amount of uncertainty was observed in the initial diagnosis of the benign group. The benign group diagnostic uncertainty rate decreased when we used the information provided by MIROR. Moreover, the false diagnosis rate for both the malignant and benign groups was reduced compared with the radiologist’s initial report with all 3 analysis methods. Additional statistical information provided to clinicians by MIROR can allow for a better and more informed noninvasive discrimination of benign and malignant body tumors in children.

**Figure 8:**
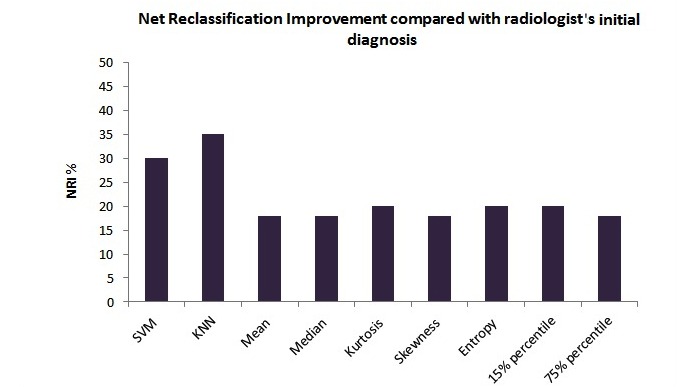
Net reclassification improvement (NRI) for k-nearest neighbor (KNN), support vector machine (SVM), and the 2-step thresholding methods compared with radiologist’s initial read.

Net reclassification improvement (NRI) [54] for KNN, SVM, and the 2-step thresholding methods using the histogram parameters were calculated to evaluate the level of improvement achieved by these methods compared with the radiologist’s initial reading . The same is presented in Figure 8. KNN had the highest incremental value (NRI=0.35) among all the methods. Histogram parameters on average had a NRI of 0.19 in comparison with the radiologist’s initial read.

## Discussion

Continuous developments of magnetic resonance systems have transformed this domain from a pure imaging system to a sophisticated precise metric system that generates a substantial amount of information and data. The complex structure of the clinical data generated often does not ease the difficulty in discriminating between different diagnoses and promotes adoption of intelligent CDSs [55,56]. CDSs allow clinicians and specialists to get insight into the data, test hypotheses, draw conclusions, and directly interact with all the available information. One should note that CDSs should aim to facilitate optimal human performance by harnessing the most advanced imaging and analysis techniques in conjunction with the end user’s own decision-making skills and abilities.

Radiologists are moving toward quantitative imaging techniques that are difficult to apply and complex to interpret [16,57]. MIROR is a real-time CDS, which can guide clinicians through the implementation and analysis of advanced and new imaging techniques and allow for these new methodologies to find clinical acceptance through translational applications.

MIROR as a diagnostic tool allows its users to extract specific region morphological features, request specific quantified metrics and features (as a biomarker), and compare with relevant findings available in its repository to gain maximal statistical power with regard to outcome prediction for the input case into the support system. MIROR can direct users to refine their search patterns looking for particular diagnoses, even if they themselves are not immediately aware of the significance of these findings.

Use of modular programming in the development of MIROR enforces logical boundaries between magnetic resonance analysis applications, thereby improving maintainability [58,59]. Modularity has also allowed development and validation of individual analysis techniques in separate studies to ensure achievement of the important feature of any CDSs, which is its accuracy and appropriateness of the system’s result.

In terms of its added clinical value and its impact on providing clinical evidence, MIROR will assist clinicians to better understand the pathophysiological difference between the different tumor types and provide information that could help them to better understand the mechanisms of diseases to improve the diagnosis and prognosis of tumors. MRI biomarkers provide information on both the tumor and its interaction with its environment and can potentially provide new information, which is not available from histology or tumor genetics. Analysis of cancer imaging big data will allow uncovering the relation and structure of cancer disease from an angle that has not previously been viewed.

Although we concentrate on developing these advanced MRI methods as a noninvasive diagnostic aid, they provide important information on tissue properties. Apparent diffusion coefficient shows a strong inverse correlation to cellularity—a key feature of tumors and tumor aggressiveness. Likewise, there is an increasing understanding that IVIM-*f* is related to tumor vascularity, which is again an important pathophysiological property of the tumor. Making these advanced MRI techniques available to clinicians in their multidisciplinary team meetings, where imaging and histopathology are evaluated together for individual cases, is an important goal and will allow an improved understanding of pathophysiology for these tumors in vivo and ex vivo.

### Limitations

As a part of future research, we plan to work on functionality and intelligent scaling of quantitative applications of MIROR by further enhancing its statistical capabilities and extension to more embedded advanced quantitative analysis modules such as magnetic resonance spectroscopy and integration of real-time interactive machine learning to optimize the use of available data in MIROR based on the guidelines [60].

It should be noted that a major limitation of the study was that MIROR was trained and tested with a rather limited set of data analyzed retrospectively. A major focus of future work will be the validation of the tool based on a prospective dataset in real time and in a multicenter clinical setting to reinforce the credibility, usability, and efficiency of the proposed CDSs applications.

### Conclusions

The proposed CDS is a diagnostic tool and repository that allows the interpretation and analysis of magnetic resonance images to be more accessible and comprehensive for clinicians. The process and experiences described here provide a model for development of the other CDSs attempting to perform a nonregion-specific quantitative analysis of MRI data. MIROR aims to increase clinician’s skillset by introducing newer techniques and up-to-date findings to their repertoire and make information from previous cases available to aid decision making. The modular-based format of the tool allowed integration of analyses that are not readily available clinically, and streamlines future developments. Pipelines for new analysis applications are available or already in development and will be shortly available under the MIROR platform.

## Abbreviations

ADC: apparent diffusion coefficient
AUC: area under the curve
CADs: computer-assisted diagnosis tools
CDS: clinical decision support
DICOM: digital imaging and communications in medicine
DWI: diffusion-weighted imaging
IVIM: intravoxel incoherent motion
KNN: k-nearest neighbor
MIROR: medical image region of interest analysis tool and repository
MRI: magnetic resonance imaging
PACS: picture archiving and communication system
ROI: region of interest
SVM: support vector machine
